# Cellular and Molecular Pathology of Age-Related Macular Degeneration: Potential Role for Proteoglycans

**DOI:** 10.1155/2016/2913612

**Published:** 2016-08-01

**Authors:** Othman Al Gwairi, Lyna Thach, Wenhua Zheng, Narin Osman, Peter J. Little

**Affiliations:** ^1^School of Health and Biomedical Sciences, RMIT University, Bundoora, VIC 3083, Australia; ^2^School of Pharmacy, The University of Queensland, Woolloongabba, QLD 4102, Australia; ^3^Faculty of Health Sciences, University of Macau, Taipa, Macau; ^4^Key Laboratory of Ophthalmology, Zhongshan Ophthalmic Center, Sun Yat-sen University, Guangzhou 510006, China; ^5^Department of Immunology, Monash University, Melbourne, VIC 3004, Australia

## Abstract

Age-related macular degeneration (AMD) is a retinal disease evident after the age of 50 that damages the macula in the centre of retina. It leads to a loss of central vision with retained peripheral vision but eventual blindness occurs in many cases. The initiation site of AMD development is Bruch's membrane (BM) where multiple changes occur including the deposition of plasma derived lipids, accumulation of extracellular debris, changes in cell morphology, and viability and the formation of drusen. AMD manifests as early and late stage; the latter involves cell proliferation and neovascularization in wet AMD. Current therapies target the later hyperproliferative and invasive wet stage whilst none target early developmental stages of AMD. In the lipid deposition disease atherosclerosis modified proteoglycans bind and retain apolipoproteins in the artery wall. Chemically modified trapped lipids are immunogenic and can initiate a chronic inflammatory process manifesting as atherosclerotic plaques and subsequent artery blockages, heart attacks, or strokes. As plasma derived lipoprotein deposits are found in BM in early AMD, it is possible that they arise by a similar process within the macula. In this review we consider aspects of the pathological processes underlying AMD with a focus on the potential role of modifications to secreted proteoglycans being a cause and therefore a target for the treatment of early AMD.

## 1. Introduction

**Figure 1:**
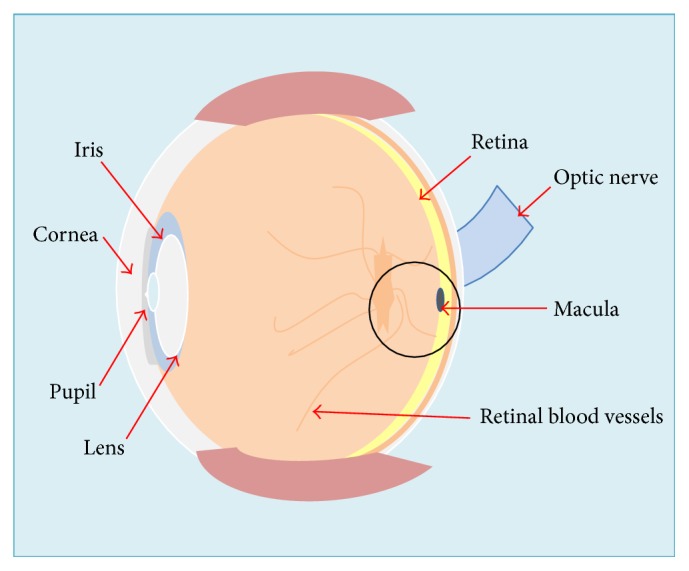
Diagram showing the anatomy of the eye including the location of the macula which is part of the retina at the back of the eye and the key site for AMD.

**Figure 2:**
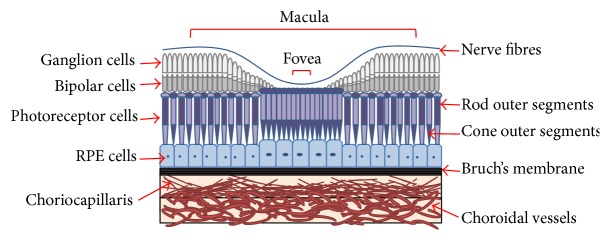
Diagram of the structure of the ocular fundus consisting of macula, fovea, photoreceptor cells, retinal pigmented epithelial (RPE) cells, Bruch's membrane, and choroidal vessels. The macula in the central retina is the key site for AMD. Two types of photoreceptors, rods and cone outer segments, and RPE cells are affected in AMD. Bruch's membrane consists of five layers and acts as a natural barrier enabling the transport of nutrients and oxygen inwards and removal of waste outwards. Choroidal vessels are lined with endothelial cells and provide the entry and exit point of nutrients and waste to the different layers of the macula.

Age-related macular degeneration (AMD) is an eye disease that develops from age 50 onwards. AMD damages the centre of the retina, specifically the macula (Figure 1), leading to a gradual visual decline [1]. It causes blurred and distorted central vision that affects visual function such as reading, driving, and face and colour recognition [2, 3]. In developed countries AMD is a common cause of blindness in the elderly [4]. AMD is ranked third globally, after cataracts and glaucoma, as the main cause of blindness [5]. In Australia, at the age of 50 and over, one in seven people are affected by AMD due to the influence of risk factors including ageing, smoking, and family history [6, 7]. The key site for initial pathological changes in AMD is the retinal barrier Bruch's membrane (BM), which is located between the retina and the choroidal circulation (Figure 2). BM plays crucial functional roles in the retina including forming a physical barrier against the invasion of new vessels, exchange of oxygen, nutrients, and waste products between the retinal pigmented epithelium (RPE) cells and the choriocapillaris, cell to cell communication, and cellular differentiation, proliferation, migration, and tissue remodelling [8, 9]. Major changes to BM in the early stages of AMD include abnormalities of the extracellular matrix components proteoglycans (PGs) and the changes can potentially increase lipoprotein to PG binding and consequently alter BM functionality [10, 11].

The early pathological features of AMD have led us and others to hypothesize that it can be considered as a disease similar in some aspects to atherosclerosis and glomerulosclerosis, which involve lipid deposition [12–15]. Previous research has focused on cell degeneration in AMD but our interest is the role of growth factors and hormones on the synthesis and structure of PGs [16, 17]. Research to date has generated some treatments for late stage AMD but there has been much less investigation of potential treatments for the early stages of AMD. The aim of this review article is to explore the role of PGs in the early stages of AMD and their potential as therapeutic targets for the prevention of AMD [14, 18, 19].

## 2. Classification of AMD

There are many classifications of AMD, all aimed at providing information about the stages of disease, but to date there is no universally accepted staging of AMD [20]. A detailed classification is available from the Age-Related Eye Disease Study (AREDS). Here we will use a simplified two-stage classification: early AMD based on the presence of drusen and indicative of early stage pathology and late AMD based on the penetration of choroidal blood vessels into the retina for wet AMD and geographic atrophy for dry AMD [2, 21, 22]. This allows us to focus on the pathological changes that are associated with either early or late BM changes.

**Figure 3:**
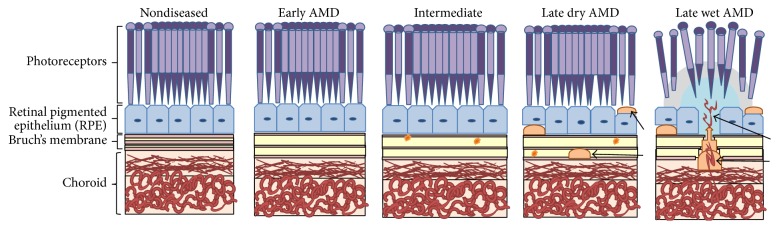
Stages of AMD: early AMD, intermediate AMD, late dry AMD, and late wet AMD. In the normal macula the arrangement of the cells is intact, Bruch's membrane is the normal shape and size, and all layers and tissues function correctly. In early AMD the tissue changes begin and a thickening of Bruch's membrane occurs. In intermediate AMD drusen (yellow circles) are clearly visible and Bruch's membrane functionality is impaired. In late AMD drusen are larger and more prominent (arrows) and cell atrophy and structural disorder occur. In wet AMD blood vessels start to grow out from the choriocapillaris and enter the macula (arrows) through Bruch's membrane.

**Figure 4:**
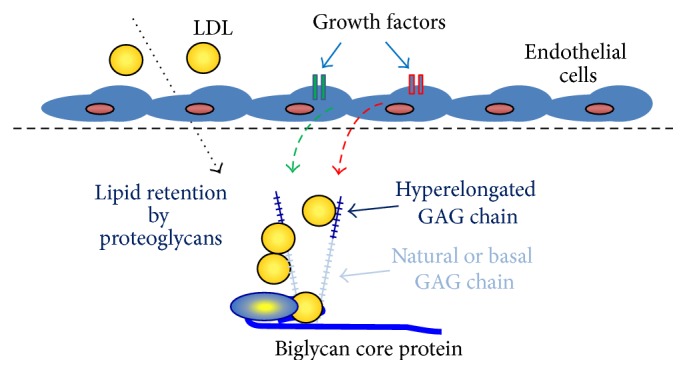
Role of growth factor mediated glycosaminoglycan (GAG) hyperelongation in the trapping of lipids in tissues. PGs have a basal or natural GAG chain length; however under the influence of growth factors the GAG synthesizing mechanism is activated leading to the increased expression of GAG elongation genes and ultimately the secretion of PGs with elongated GAG chains. These chains have increased binding to LDL (see text for details). Lipids trapped in tissues undergo chemical modifications which produce immunogens that initiate an inflammatory cascade and ultimately a chronic inflammatory disease process.

There are two types of drusen: large, soft drusen associated with early AMD [22] and small hard drusen typically seen with increasing age and not directly related to the development of AMD. Choroidal neovascularization (CNV) starts from the choroidal vasculature with leaky blood vessels breaking through BM into the RPE cell layer. CNV can result in the leakage of blood and accumulation of serum in the eye leading to irreversible vision loss in a short period of time [21, 23–25] (see Figures 3 and 4).

Dry AMD, or non-CNV AMD, is also associated with the formation of drusen. They contain lipid, proteins, and undigested RPE waste deposited outside the RPE cell layer. Dry AMD has three stages: early, intermediate, and advanced. The early stage is characterised by an accumulation of insoluble extracellular substances which later form round, yellow, well demarcated small to medium size drusen, less than 64 *µ*m in diameter [21, 22, 26]. Geographic atrophy (GA) denotes the late stage of dry AMD and is characterised by a significant loss of photoreceptor cells and in many cases involves atrophy of RPE cells within GA areas of the macula [27]. About 65% of the patients with dry AMD can develop wet AMD. Although wet AMD accounts for only 8% of AMD cases, it is responsible for 85% of vision loss. Dry AMD is considered a risk factor for wet AMD [28].

Progression of AMD shows wide variation among patients and is characterised by many factors related to the early signs of AMD; these include the extent of geographic atrophy, RPE abnormalities, pigment changes, and the declining rate of the reading speed [29] as noted by the AREDS study [30–32]. The alteration of extracellular matrix components leads to an increased deposition of lipids and protein components in BM. This diffuse thickening of BM when seen in conjunction with the presence of drusen is considered a clinical hallmark for AMD and further abnormalities can quickly shift the disease to the CNV stage of development [33, 34].

## 3. Risk Factors for AMD

AMD has other strongly associated, weakly associated, and under-investigation risk factors [2, 6, 30, 32, 35–39]. A strong environmental risk factor is smoking. Smokers have three times the risk of developing AMD [2, 35]. Ageing is also a strong risk factor with a strong increase in prevalence and progression of AMD with age. It has been reported that the older the patients, the later their stage of AMD. Genetic factors indicated by family history of AMD are also a strong risk factor with a family history of AMD predicting that a person will have a 50% chance of developing AMD and a particularly strong association with advanced AMD [35, 40].

Weaker risk factors for AMD include body mass index (BMI), cardiovascular disease, hypertension and elevated plasma fibrinogen, gender (females are more susceptible than males), ethnicity (light skin colour is more prone to develop AMD), diabetes, serum lipid levels, and exposure to sunlight (increases the risk of AMD twofold) [6, 21]. Notably for this review there is an association between AMD and serum lipids levels but relatively not much attention has been given to this observation [41, 42]. Emerging risk factors for AMD include diet composition, that is, vitamins, minerals, antioxidants, and cholesterol levels, and also lifestyle factors such as alcohol consumption, obesity, and physical activities [39, 43]. There is inconsistency in the relationship between cataracts and AMD as some research supports a relationship and others have found none. However there is thought to be a relationship between AMD and hyperopia, as well as hormonal levels and AMD [6].

## 4. Pathology of the Macula in the Development of AMD

The ocular fundus of the eye consists of five parts, the macula, retina, optic disc, fovea, and posterior pole, and includes BM which is part of the choroid (see Figure 3) [7, 44]. Of the two types of photoreceptors in the eye rod cells support vision at low light levels and are used in the peripheral vision. On the other hand, the cones function best in bright light and are responsible for colour and central vision. The macula contains far more cone photoreceptors in comparison to the rest of the retina. Thus it plays a vital role in central vision and acuity and enhances the resolution of details. The macula is the most affected part of the eye by AMD [21]. The outer retina consists of a multilayer network of interdependent cells and contains the retinal blood barrier which is the highest oxygen consumer in the human body (weight to consumption ratio). This retinal blood barrier has the property of selective permeability and it is immune privileged [45].

Defects, damage, or disorder to BM can lead to a loss of its functions and one of the most important is the transit of waste from RPE cells and photoreceptors to exit to the choroidal blood vessels. In AMD early altered function can be identified as a thickening of BM. Drusen formation is a main characteristic of early AMD. Histologically drusen are focal deposits between the basal lamina of the RPE and the inner collagenous layer of BM [46, 47]. They contain proteins, lipoproteins, including apoB, apoE, and lipids cholesterol, and phosphatidylcholine [47, 48]. Drusen originate from a mixture of RPE membrane debris, active lipid release from RPE, and some plasma lipid [45]. Nonfocal AMD laminar deposits include BLinD and BLamD. BLinD are similar in content and site of deposition as drusen; however they form thin layers, are an early thickening of BM, and along with soft drusen are more commonly found in the central macula [49]. BLamD are found alongside BM at the RPE basal lamina and contain cholesterol and other lipoproteins [45]. The formation of drusen happens over a period of many years [7, 9, 21]. In nondiseased aged macula small drusen can exist without significant effect [50, 51]; however numerous larger drusen are more commonly associated with AMD [52, 53]. Other progressive features of AMD include BM thickening, geographic and RPE atrophy, degeneration of BM and RPE, and CNV.

Altered BM thickness and function could result from increased lipid deposition as a consequence of positively charged lipids and lipoproteins binding to negatively charged PGs and thereby impacting BM function. PG-mediated lipid deposition is a key event in early atherosclerosis and altered PG synthesis and structure such as increased glycosaminoglycan chain sulfation status and/or length result in an increased lipoprotein to PG binding ratio and greater deleterious lipid deposition [14, 18, 45, 54–56]. The extent to which this occurs in AMD is still not clear. CNV is the end result of more than 40 described pathological processes and is the diagnostic feature of wet AMD. BM plays a vital role as a shield or barrier against the penetration of capillary blood vessels into the retina and the subsequent progression of CNV [8].

## 5. Molecular Pathogenesis of AMD

Based on the current understanding of AMD there are many pathological processes that contribute to AMD at the molecular and biochemical levels. These include oxidative damage, abnormal lipid metabolism, apoptosis, structural abnormalities of photoreceptor outer segments, dysfunction of ion channels in the RPE, variations in the immune system, and abnormalities of the extracellular matrix [45, 57, 58]. The extracellular matrix composition is varied throughout the retina and any alteration, that is, becoming depleted, new synthesis, or increased decomposition and waste, can lead to AMD-related retinal changes. For example, the retina contains high concentrations of lutein and zeaxanthin, both macula pigments that function as antioxidant and blue light filters that protect the retina from photooxidative damage and can halt the progression of AMD [32, 39]. Lysis of intracellular material may be converted to waste in the extracellular matrix [28, 43, 44, 59]. In view of the role of PGs in lipid retention in atherosclerosis, it may prove useful to study the role of growth factor signalling to PGs in the development of early BM changes [60–62].

## 6. Proteoglycan Structure and Alterations in AMD

Growth factors and hormones stimulate cells to cause the elongation of glycosaminoglycan (GAG) chains on PGs which leads to enhanced lipid binding and the trapping of material in tissues (see Figure 4) [10, 63]. The function of PGs varies greatly depending on the core protein family and the type, number, size, and sulfation of GAG chains. Generally PGs act as a binder for the extracellular matrix as well as a pool for growth factors and tissue hydration [63]. PGs are classified into four major families: heparan sulfate (HS), chondroitin sulfate (CS), dermatan sulfate, and keratan sulfate [64]. Glycosaminoglycan chains are linear polysaccharides that are attached in variable numbers to PG core proteins [55, 65].

PGs can be present on the cell surface and are also secreted into the extracellular matrix of mammalian tissues [63, 64]. Prominent PG species in the eye include the small leucine-rich CSPGs decorin and biglycan and it is an established paradigm that tissue insult leads to their increased production [28, 57]. PGs are heavily sulfated and thus negatively charged and they bind and retain positively charged molecules including apolipoproteins on the surface of lipids [66]. The specific role of PGs in the development and progression of AMD has not yet been fully explored despite their key role in extracellular matrix deposition in the thickening of Bruch's membrane in early AMD and their facilitation of neovascularization in wet AMD. Bruch's membrane is acellular and dependent on the adjacent RPE cells for the production of its extracellular matrix components including PGs which provide Bruch's membrane with an overall negative charge. The discontinuous nature of the elastic lamina of Bruch's membrane in the macula highlights the potential of choroidal cells including endothelial, fibroblast, and vascular smooth muscle cells to also contribute matrix components including PGs. In 1989 Tyl Hewitt et al. [54] using whole eye organ cultures showed that the proportion of newly synthesized PGs remains consistent at 75% chondroitin/dermatan sulfate (CS/DS) and 25% heparan sulfate (HS). CS/DS GAGs are associated with collagen fibrils in Bruch's membrane and HS is found near the basement membrane of RPE layer and choriocapillaris [20]. In 2012 Ambati and Fowler [21] showed that AMD extracts express CS and HS GAGs. Total GAG content is significantly higher in maculae containing BLamD. The relative contribution of HSPGs and CSPGs to the pathogenesis of AMD is not well understood.

HSPGs play vital role in AMD through regulation of the complement system. HS is a long unbranched polysaccharide glycosaminoglycan that is attached to ocular PG core proteins in the extracellular matrix of BM and macular tissues [63, 64]. HS chains are variable in length and sulfation and it has been shown that the quantity and sulfation of HS within BM decrease with age [67]. This impacts the progression of AMD because there are likely to be fewer binding sites for complement factor H (FH) a known risk factor for AMD particularly when individuals have the Y402H polymorphism [68, 69]. FH negatively regulates and prevents activation of complement.

## 7. Role of Growth Factors and Their Signalling Pathways

Vasoactive growth factors are known to regulate PG synthesis and structure; hence their signalling pathways are potential therapeutic targets for lipid deposition diseases such as atherosclerosis and glomerulosclerosis [70]. We know from our studies that in vascular smooth muscle a number of growth factors increase the synthesis of CSPGs and glycosaminoglycans including platelet-derived growth factor, vascular endothelial growth factor, transforming growth factor beta, insulin-like growth factor, and thrombin [60–62, 66, 71]. CSPGs via their GAG chains bind lipid and cause lipid deposition in artery walls and promote atherosclerosis. Currently studies are underway to investigate the contribution of these growth factors to the early stages of AMD. It is interesting that HSPGs and sulfation are reduced with age [67]; this suggests a reduction in lipid binding and less lipid deposition in BM. However it is possible that, in contrast to HSPGs, the CSPGs and their glycosaminoglycan chains could be longer and/or more sulfated in AMD since the enzymes that regulate the HSPGs and CSPGs chain synthesis differ and hence the effects of different growth factors may also be different.

## 8. Proteoglycan Lipid Interactions

It has been established that the extracellular matrix is very important in controlling many biological processes that lead to the progression of AMD. The length of endogenously secreted GAG chains is such that increases in length produce a large increase in the interaction between PGs and lipids [45, 72]. Increased interactions between PGs and lipids lead to increased accumulation of lipids in the retina [73]. One of the most important lipids in AMD is cholesterol with high levels of cholesterol in the lipid-rich drusen associated with AMD. It will be beneficial to explore the possibility that these deposits might arise by the trapping of lipids by PGs with elongated GAG chains and the impact such interactions have on the pathogenesis of AMD [48].

## 9. Conclusions

It is clear that more research into the early phase of AMD is needed to target early changes in the macula as most works have focused on the later stages of the disease process. Whilst approximately 15% of cases are wet AMD, 85% of cases are the dry form of AMD which has no available treatments. Treatments for wet AMD do not improve dry AMD [21]. This suggests that research on early AMD might yield new treatment targets. On the basis that there is evidence for the accumulation of plasma derived lipids in early AMD it is reasonable to propose that the trapping of lipids by modified PGs might underlie the disease process in the early stages of AMD; thus detailed studies of the metabolism of PGs by all retinal cells are warranted in the search for a new therapeutic target in an area in which none currently exists.
